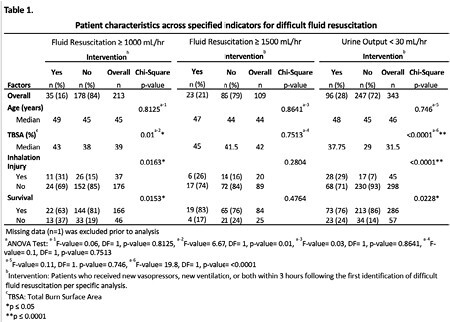# 8 Burn Shock: What Defines a Failing Fluid Resuscitation?

**DOI:** 10.1093/jbcr/irae036.008

**Published:** 2024-04-17

**Authors:** Abigail Plum, Ryan M Johnson, Kevin E Galicia, John Kubasiak

**Affiliations:** Loyola University Chicago, Oak Park, Illinois; Loyola University, Maywood, Illinois; Loyola University Chicago, Chicago, Illinois; Loyola University Chicago, Oak Park, Illinois; Loyola University, Maywood, Illinois; Loyola University Chicago, Chicago, Illinois; Loyola University Chicago, Oak Park, Illinois; Loyola University, Maywood, Illinois; Loyola University Chicago, Chicago, Illinois; Loyola University Chicago, Oak Park, Illinois; Loyola University, Maywood, Illinois; Loyola University Chicago, Chicago, Illinois

## Abstract

**Introduction:**

Major burn injuries often elicit burn shock requiring acute fluid resuscitation for patient survival. Successful resuscitation is a significant challenge for patient management as under-resuscitation and over-resuscitation can lead to greater adverse events. At the ABA-State of the Science in 2021, proposed definitions included >1,500ml IVF per hour, although no clear clinical data supported this expert consensus. A clear definition of a failing resuscitation may better guide physician decision-making for additional interventions. The primary objective of this study was to examine the association between intervention provided within three hours following a defined indication of failing resuscitation and patient survival.

**Methods:**

The study utilized the Acute Burn ResUscitation Multicenter Prospective Trial (ABRUPT), consisting of patients ≥18 years with burns ≥20% of total body surface area (TBSA), to examine three indications of failing resuscitation. Three mutually exclusive analyses were conducted on patients that had fluid resuscitation of ≥1000 mL in one hour, fluid resuscitation of ≥1500 mL in one hour, or two consecutive hours of urine output < 30 mL. Intervention was defined as the patient having new vasopressors or a new ventilator within three hours of the first indication of failing resuscitation. Multivariable logistic regression models were used to assess the associations of interest and to adjust for confounders.

**Results:**

Patients with a failing resuscitation indicator of ≥1000 mL/hr who received an intervention were 46% more likely to be alive at the end of the study (OR=0.54: 95% CI=0.22-1.33). Patients with a failing resuscitation indicator of ≥1500 mL/hr who received an intervention were 50% less likely to be alive at the end of the study (OR=1.5: 95% CI=0.47-5.01). Patients with a failing resuscitation indicator of urine output < 30 mL who received an intervention were 6% less likely to be alive at the end of the study (OR=1.06: 95% CI=0.51-2.22). These results were adjusted for age, TBSA %, and inhalation injury.

**Conclusions:**

A lower indication for failing resuscitation (≥1000 mL/hr) followed by an intervention shows potential for increased patient survival, although adjusted analyses were insignificant likely due to the small patient sample size. Further work is needed to conclude a sufficient definition of failing resuscitation for burn shock.

**Applicability of Research to Practice:**

Identifying a clear definition of failing resuscitation will serve as a consistent guide for physician response to burn shock.